# Dexmedetomidine as a promising prevention strategy for cardiac surgery-associated acute kidney injury: a meta-analysis

**DOI:** 10.1186/s13054-017-1776-0

**Published:** 2017-08-03

**Authors:** Rui Shi, Hong-Tao Tie

**Affiliations:** 1 Department of Cardiology, The First Affiliated Hospital of Chongqing Medical University, Chongqing, 400016 China; 2 Department of Cardiothoracic Surgery, The First Affiliated Hospital of Chongqing Medical University, Chongqing, 400016 China

**Keywords:** Cardiac surgery, Acute kidney injury, Dexmedetomidine, Meta-analysis

Dexmedetomidine has a possible protective effect on cardiac surgery-associated acute kidney injury (CSA-AKI); however, current evidence is limited and controversial. We therefore conducted a meta-analysis regarding dexmedetomidine for CSA-AKI.

PubMed and EMbase were searched. A random-effects model in RevMan 5.3 software was used, and *P* < 0.05 indicates statistical significance. Three randomized controlled trials (RCTs) with 338 patients and four cohort studies involving 19,266 participants were included. The main characteristics are shown in Table 1. Overall results show that dexmedetomidine was associated with a significantly reduced incidence of CSA-AKI in both the RCTs (relative risk [RR] 0.44, 95% confidence interval [CI] 0.26–0.76, *p* = 0.003) and cohort studies (RR 0.74, 95% CI 0.63–0.86, *p* = 0.0001) (Fig. 1) without significant heterogeneity (RCT *I* ^*2*^ = 0%; cohort *I* ^*2*^ = 0%). For secondary outcomes, dexmedetomidine failed to decrease postoperative mortality (RCT RR 0.20, 95% CI 0.02–1.68; cohort RR 0.56, 95% CI 0.28–1.15), duration of mechanical ventilator (RCT standard mean differences [SMD] −0.18, 95% CI −2.08–1.71; cohort SMD −0.12, 95% CI −0.25–0.01), intensive care unit stay (RCT SMD −0.21, 95% CI −0.53–0.11; cohort SMD −0.52, 95% CI −1.06–0.02), and hospital length of stay (SMD −0.34, 95% CI −1.21–0.54). However, decreased trends were observed for all secondary outcomes.

**Table 1:** Main characteristic of the seven included studies

StudyID	Study type	Number (DEX/Control)	Surgery type	Intervention		Ref (DOI)
				DEX	Control	
Ammar et al. 2016 [4]	RCT	25/25	Cardiac surgery with CPB	5 min before CPB until 6 h after surgery (1 μg/kg for 15 min and followed by 0.5 μg/kg/h)	Placebo	10.4103/1658-354X.177340
Balkanay et al. 2015 [2]	RCT	60/28	CABG with CPB	After ICU admission and continuing for a maximum of 24 h (0.04 μg/kg/h to 0.5 μg/kg/h)	Placebo	10.1093/icvts/ivu367
Cho et al. 2015 [5]	RCT	100/100	Cardiac surgery with CPB	After anesthetic induction and continuing for 24 h after surgery (0.4 μg/kg/h)	Placebo	10.1038/ki.2015.306
Ji et al. 2013 [3]	Cohort (retrospective)	567/566	CABG/valve surgery with CPB	After CPB and continuing for a maximum of 24 h (0.24 μg/kg/h to 0.6 μg/kg/h)	Control	10.1371/journal.pone.0077446
Kwiatkowski et al. 2016	Cohort (retrospective)	102/102	Cardiac surgery with CPB	NR	Control	10.1097/PCC.0000000000000611
Shehabi et al. 2012	Cohort (prospective)	76/77	Cardiac surgery with CPB	After anesthetic induction and until extubation (0.7 μg/kg/h)	Control	10.1097/01.ccm.0000425199.76669.9f
Turan et al. 2014	Cohort (retrospective)	765/17,011	Cardiac surgery	NR	Control	10.1016/j.jclinane.2014.05.009

*CABG* coronary artery bypass graft, *CPB* cardiopulmonary bypass, *DEX* dexmedetomidine, *ICU* intensive care unit, *NR* not reported, *RCT* randomized controlled trial

**Fig. 1:**
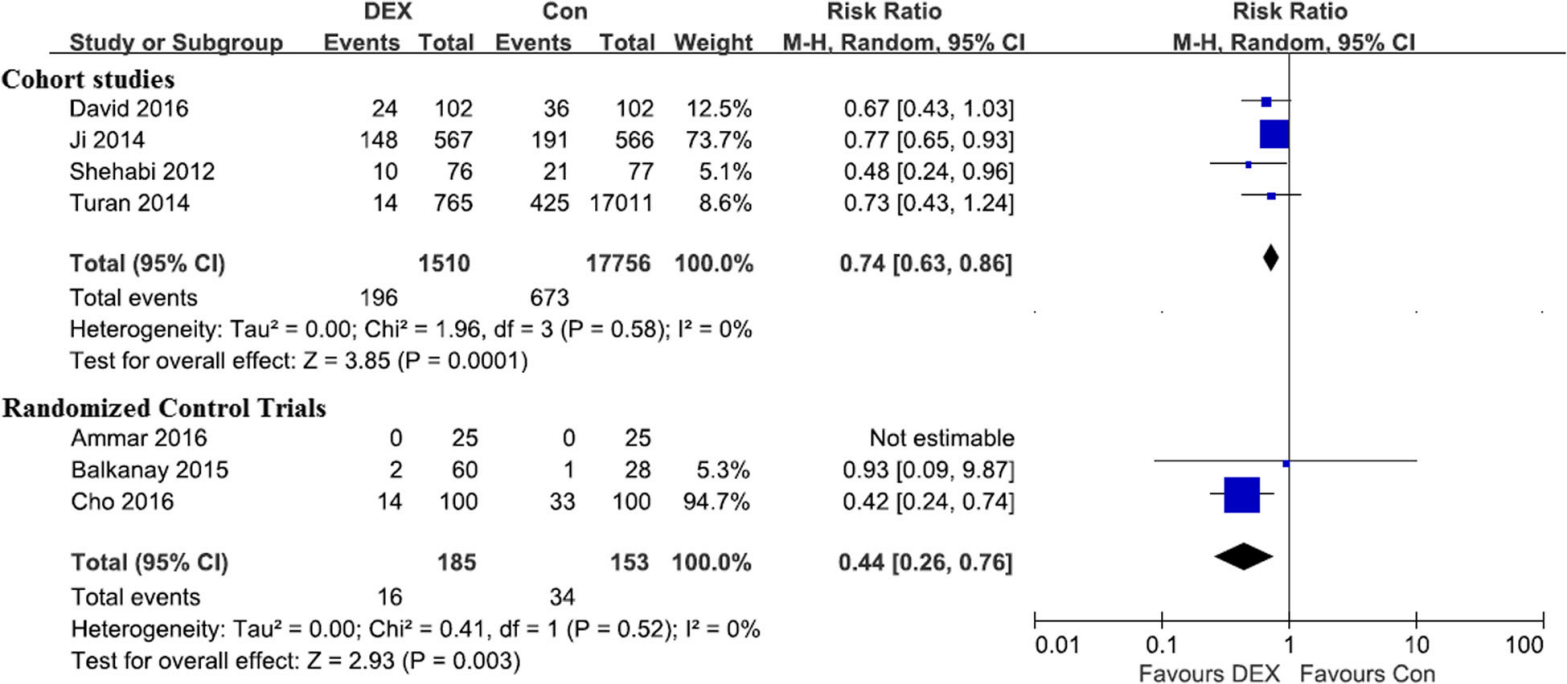
Forest plots for the meta-analysis of dexmedetomidine and the incidence of CSA-AKI

A retrospective cohort study [1] and an RCT [2] were not consistent with the other included studies in our meta-analysis. This inconsistency could be explained by limitations of retrospective studies, different CSA-AKI criteria, different doses and duration of dexmedetomidine for the cohort, and CSA-AKI criteria for the RCT because the preventive effect was found when defined by NGAL concentration but not RIFLE classification.

The underlying mechanism is multifactorial, and current evidence demonstrates that, as a selective α2-adrenoreceptor agonist, the renoprotective function of dexmedetomidine could be achieved by promoting renal blood flow via inhibiting vasoconstriction and promoting a diuresis effect via decreasing renin and arginine vasopressin and increasing glomerular filtration [3]. Additionally, protection from kidney ischemia/reperfusion injury by reducing reactive oxygen species, decreased systemic inflammatory response, and reduced renal cell death in cardiac surgery were also involved [4].

Hypotension and bradycardia caused by dexmedetomidine are often of concern, mainly with loading and maintenance doses >0.7 μg/kg/h [5]. All reported dexmedetomidine doses were lower than 0.7 μg/kg/h in our meta-analysis except for two unknown cohorts. Additionally, dexmedetomidine’s safety and efficacy have been confirmed in cardiac surgery [1].

In summary, dexmedetomidine might be a promising prevention strategy for CSA-AKI. More high-quality RCTs are encouraged to verify the beneficial effect of dexmedetomidine before its clinical application.

## Abbreviations

CI: Confidence interval
CSA-AKI: Cardiac surgery-associated acute kidney injury
ICU: Intensive care unit
RCT: Randomized controlled trial
RR: Relative risk
SMD: Standard mean difference
